# Norwalk Virus Shedding after Experimental Human Infection

**DOI:** 10.3201/eid1410.080117

**Published:** 2008-10

**Authors:** Robert L. Atmar, Antone R. Opekun, Mark A. Gilger, Mary K. Estes, Sue E. Crawford, Frederick H. Neill, David Y. Graham

**Affiliations:** Baylor College of Medicine, Houston, Texas, USA

**Keywords:** norovirus, gastroenteritis, RT-PCR, Norwalk virus, adult, human, research

## Abstract

Noroviruses are the most common cause of viral gastroenteritis in the United States. To determine the magnitude and duration of virus shedding in feces, we evaluated persons who had been experimentally infected with Norwalk virus. Of 16 persons, clinical gastroenteritis (watery diarrhea and/or vomiting) developed in 11; symptomatic illness lasted 1–2 days. Virus shedding was first detected by reverse transcription–PCR (RT-PCR) 18 hours after participant inoculation and lasted a median of 28 days after inoculation (range 13–56 days). The median peak amount of virus shedding was 95 × 10^9^ (range 0.5–1,640 ×10^9^) genomic copies/g feces as measured by quantitative RT-PCR. Virus shedding was first detected by antigen ELISA ≈33 hours (median 42 hours) after inoculation and lasted 10 days (median 7 days) after inoculation. Understanding of the relevance of prolonged fecal norovirus excretion must await the development of sensitive methods to measure virus infectivity.

## Abstract 2

Noroviruses are shed in feces up to 8 weeks after infection.

Noroviruses are the most common cause of epidemic gastroenteritis in the United States (*1*). Although in vitro replication systems for these viruses have recently been described (*2*,*3*), human noroviruses cannot readily be grown in cell culture, and no small animal model of human norovirus infection is available. Much of what is known about these viruses has by necessity been learned from experimental human infection and from observational studies of naturally acquired infection. Norwalk virus is the prototype strain in the genus *Norovirus*, and many of the human experimental infection studies have used this strain (*4*–*9*). We describe the duration and magnitude of virus shedding in persons infected with Norwalk virus after experimental inoculation.

## Materials and Methods

### Virus Inoculum

Liquid feces from persons who participated in a previous Norwalk virus challenge study (*8*) were screened to identify samples with high concentrations of Norwalk virus RNA (>10^7^ reverse transcription–PCR [RT-PCR] U/mL). The participants were recontacted and screened for health (results within normal limits for liver function, tuberculosis skin test [negative], and chest radiographs; negative serologic test results for hepatitis A, B, and C, retroviruses [HIV-1, HIV-2, and human T-lymphotropic virus 1 and 2], and syphilis [nonreactive rapid plasma regain]). The new challenge inoculum (lot 42399) was prepared from liquid feces of 1 participant by clarification, centrifugation, and serial filtration through filters with progressively smaller pore size to a final 0.45-μm filter size. The inoculum, which contained no other enteric viruses or adventitious agents, was packaged and stored at –80°C.

### Challenge Protocol

Challenge studies were conducted from September 2004 through October 2006. Healthy adults (18–50 years of age) provided informed consent and successfully completed a test of understanding. In addition, eligible persons were secretor positive (because secretor-negative persons are resistant to Norwalk virus infection; *9**,**10*), had screening laboratory study results that were within normal limits (liver and renal function, blood counts), had negative serologic results for hepatitis and HIV, had no serious chronic diseases, had no history of nonbacterial gastroenteritis within 3 months of inoculation or of bacterial or protozoal enteric infection within 1 month (based on 3 negative enteric cultures and fecal ova and parasite studies in the 4-week preinoculation screening period), were not exposed to persons considered to be at risk for more severe norovirus infection (e.g., immunocompromised patients, the elderly, and children), and were not employed in jobs identified as having high risk for transmission to other persons (e.g., food service, healthcare, and airline industries). On the day of inoculation, participants were admitted to the Baylor College of Medicine General Clinical Research Center and orally received different dosages of inoculum (10-fold dilutions ranging from 4.8 to 4,800 RT-PCR units) or placebo in the early evening. Inoculated participants remained in the Center for a minimum of 96 hours and at discharge had experienced no watery feces or vomiting for at least 18 hours. Participants’ clinical signs and symptoms were evaluated every 4 hours while they were in the Center, and all fecal samples were collected and refrigerated immediately. Within 24 hours of collection, the samples were transported to the laboratory for processing and stored at –70^o^C until analyzed. After patient discharge, all fecal samples were collected daily for 21 days and then weekly for up to 5 additional weeks (for a total observation time of up to 8 weeks postinoculation). The samples were delivered to the laboratory within a day of collection and were processed and stored as described above. Participants were educated about the importance of hand washing and hand hygiene at the beginning of the study, and these concepts were reinforced at each study visit. The clinical protocol was reviewed and approved by the Institutional Review Board at Baylor College of Medicine, and an Investigational New Drug application describing the clinical protocol and study inoculum was reviewed by the US Food and Drug Administration.

### Laboratory Studies

Norwalk virus–specific antigen was detected by sandwich ELISA, using Norwalk virus–specific antiserum, as previously described (*8*), and Norwalk virus–specific antibody was detected by ELISA, using Norwalk virus–like particles as antigen, as previously described (*8*). Norwalk virus RNA was detected in fecal specimens by using either an immunomagnetic capture (IMC) RT-PCR assay (*11*) or quantitated by real-time RT-PCR (qRT-PCR) with RNA transcripts as a standard (*2*). The primers used for the IMC RT-PCR assay were the antisense Norwalk virus p35 (5′-CTT GTT GGT TTG AGG CCA TAT-3′) and the sense Norwalk virus p36 (5′-ATA AAA GTT GGC ATG AAC A-3′); probe was a 5′ digoxigenin-labeled Norwalk virus p69 (5′-GGC CTG CCA TCT GGA TTG CC-3′). For the qRT-PCR assay, a 10% fecal sample was diluted 1,000-fold and heated to 95^o^C for 5 min (*12*); 20 μL of heated sample was analyzed in duplicate wells. The primers for the qRT-PCR assay were the antisense Norwalk virus p165 (5′-CAT AAT CAC CTA CAT CCA TCT CAG ATG-3′, which is complementary to Norwalk virus nt 4689–4715) and the sense primer Norwalk virus p166 (5′-CGG CCT CAC CAG AAT TGG-3′, which is complementary to Norwalk virus nt 4641–4658); the probe was Norwalk virus p167 (5′-FAM/CGA GGT TGT GGC CCA AGA TTT GCT AG/TAMRA-3′, which is complementary to nt 4660–4685). For determination of a virus titer, both wells had to show amplification. The limits of detection for the IMC RT-PCR and qRT-PCR assays were ≈15 × 10^3^ and ≈40 × 10^6^ copies/g feces, respectively.

### Definitions

Infection was defined as seroresponse (4-fold rise in titer from preinoculation baseline to 30-day serum sample, as measured by ELISA) or fecal virus excretion as detected by RT-PCR or presence of antigen. Viral gastroenteritis was defined as illness with moderate diarrhea (alone) for any continuous 24-hour period (moderate diarrhea is >200 g of watery feces that immediately take the shape of the collection container) or 1 vomiting episode plus 1 of the following: abdominal cramps or pain, nausea, bloating, loose feces (if not fulfilling the definition of diarrhea), fever (oral temperature >37.6°C), myalgia, or headache.

## Results

A total of 16 persons inoculated with Norwalk virus met the criteria for having Norwalk virus infection. Of these, 11 (69%) met the predefined definition for viral gastroenteritis. The 5 who did not meet this predefined definition had >3 symptoms that did not include vomiting or >200 g of watery diarrhea. All 11 participants with viral gastroenteritis had abdominal cramps, nausea, and vomiting; 5 of these participants also had >200 g of watery diarrhea, and 1 had <200 g of watery feces. Other signs and symptoms in the 11 participants were malaise (n = 9), anorexia (n = 8), headache (n = 7), myalgia (n = 4), temperature >37.6^o^C (n = 4), and chills (n = 3). The 5 participants who did not fulfill the criteria for gastroenteritis had nausea (n = 5), anorexia (n = 5), malaise (n = 4), abdominal cramps (n = 3), myalgia (n = 3), headache (n = 3), temperature >37.6^o^C (n = 2), chills (n = 2), and watery diarrhea <200 g (n = 2). Although the number of infected participants in each dosage group was relatively small, no differences in signs and symptoms based on inoculum dosage were apparent. The median duration of signs and symptoms was 23 (range 10–61) hours and was similar for both groups of participants.

All infected participants shed virus as measured by RT-PCR and had a >4-fold rise in serum antibody level, and all but 2 also shed virus as measured by antigen ELISA (Table). Virus shedding as measured by IMC RT-PCR was first detected a median of 36 hours (range 18–110 hours) after inoculation and lasted a median of 28 days after inoculation (range 13–56 days). Norwalk virus was detected in fecal samples of 7 participants 3–14 hours before onset of any clinical signs or symptoms. Presymptomatic shedding was more common in persons who did not meet the definition of clinical gastroenteritis than in those who did (4/5 vs. 3/11, respectively, p = 0.11, 2-tailed Fisher exact test). Virus shedding as measured by antigen ELISA was first detected ≈33 hours (median 42 hours) after inoculation and was last detected 10 days (median 7 days) after inoculation. Median values of the onset and resolution of virus shedding, as measured by IMC RT-PCR or antigen ELISA, were similar for the participants who had clinical gastroenteritis compared with those of persons who did not meet the definition of gastroenteritis (Table).

**Table:** Fecal virus shedding among 16 participants inoculated with Norwalk virus*

Participant no.	Estimated Norwalk virus inoculum dose (RT-PCR units)	First–last study days† postinoculation when symptoms present	First–last study days IMC RT-PCR positive	Day peak virus titer (character of feces)	Peak qRT-PCR virus titer (log_10_/g)	First–last study days postinoculation when antigen positive
Met clinical definition of gastroenteritis						
Had diarrhea and vomiting						
706	4,800	2	2–28‡	2 (liquid)	11.1	2–9
707	4,800	2–4	1–30‡	2 (liquid)	9.5	4–8
710	4,800	1–2	2–30‡	5 (solid)	10.9	2–7
722	48	2	2–48	4 (solid)	11.7	2–8
724	4.8	2–3	2–56	3 (solid)	11.4	2–6
Had vomiting only						
701	4,800	1–2	1–29‡	4 (solid)	10.8	3–10
720§	48	2	2–56	4 (solid)	11.5	2–9
721	48	1–3	2–21	4 (solid)	11.7	2–10
723	48	2	1–50	4 (solid)	12.2	2–6
731	4.8	2–3	5–10	5 (solid)	10.0	None
732	4.8	2–3	2–15	3 (solid)	11.9	2–6
Median	–	2	2–30‡	4 (solid)	11.4	2–8
Did not meet clinical definition of gastroenteritis						
703	4,800	2–3	1–32‡	2 (solid)	10.7	2–9
704	4,800	2–3	4–21‡	5 (solid)	9.2	5–7
715§	48	2–3	1–28	3 (solid)	11.7	2–5
716§	48	2–3	1–20	4 (unformed)	10.1	3–7
717	48	3	4–13	4 (solid)	9.3	None
Median	–	2–3	1–21‡	4 (solid)	10.1	2–7

*RT–PCR, reverse transcription–PCR; IMC, immunomagnetic capture; qRT-PCR, quantitative PCR. †Study days are reported as calendar days; study day 1 began ≈5–6 h postinoculation. ‡Fecal samples only collected for 30 d postinoculation. §Watery feces with mass <200 g.

**Figure 1:**
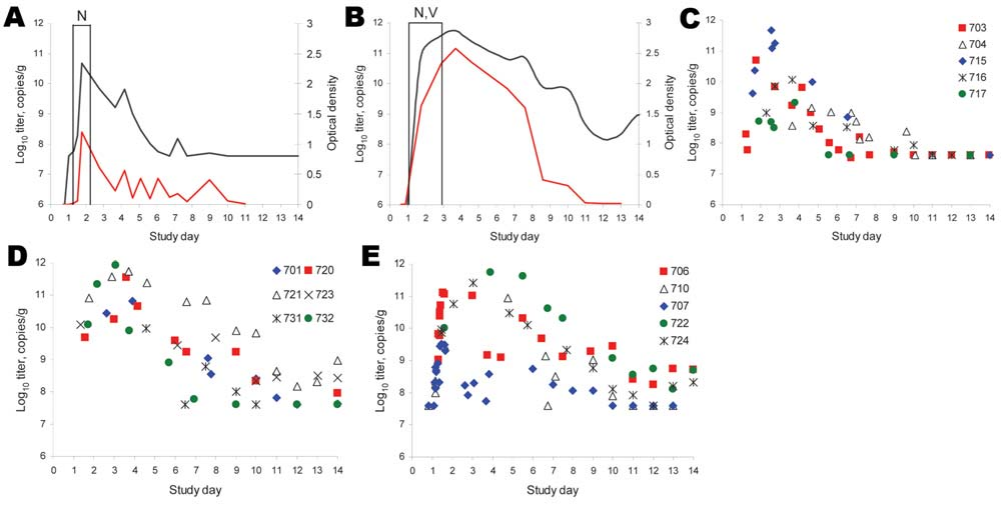
Shedding of Norwalk virus in feces. The quantity of viral RNA measured by quantitative reverse transcription–PCR (qRT-PCR; black line) and of virus antigen measured by ELISA (optical density; blue line) is shown for 2 participants: no. 703, who did not have clinical gastroenteritis (panel A), and no. 721, who had clinical gastroenteritis (panel B). Vertical lines represent the period of clinical symptoms; N, nausea; V, vomiting. Panels C, D, and E show the virus titers as measured by qRT-PCR in fecal samples collected from participants who had no clinical gastroenteritis, had gastroenteritis with vomiting only, and had gastroenteritis with vomiting and diarrhea, respectively.

**Figure 2:**
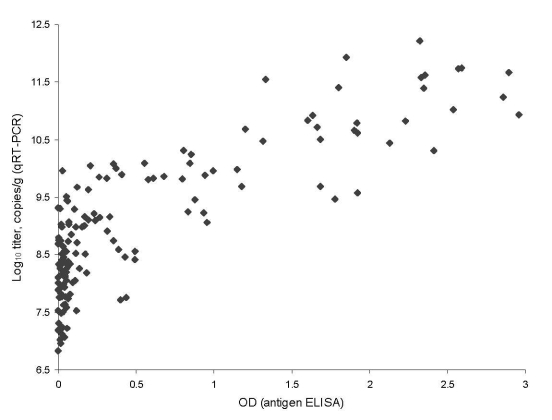
Correlation of viral RNA titer with antigen ELISA (optical density [OD]). Titers of viral RNA are correlated with the OD measured by antigen ELISA for the 148 fecal samples with positive quantitative reverse transcription–PCR (qRT-PCR) results (r^2^ = 0.823, Spearman correlation, p<0.001).

Norwalk virus concentration in feces, as measured by qRT-PCR, peaked a median of 4 days after inoculation; the time of peak shedding was similar for participants who did and did not meet the definition of viral gastroenteritis (Table). The highest fecal concentrations of virus were detected in 11 (69%) participants after their clinical signs had resolved. The median peak amount of virus shedding was 95 × 10^9^ (range 0.5–1,640 × 10^9^) genomic copies/g feces, and 5 participants shed >100 × 10^6^ copies/g until at least day 14 (Figure 1). Persons who met the clinical definition of gastroenteritis had a higher median peak of virus shedding than those who did not have gastroenteritis (250 × 10^9^ vs. 12 × 10^9^ genomic copies/g feces, p = 0.08, Wilcoxon rank sum), and the average total number of viral genomic copies measured in the feces over the first 2 weeks after inoculation also was higher in the clinical gastroenteritis group (10^13.3^ vs. 10^12.4^, p = 0.056, Student *t* test). The virus concentrations in feces collected later after inoculation were low (range 225,000–40 × 10^6^ genomic copies/g). Correlation between virus titer in feces and optical density results obtained in the antigen ELISA was strong (r = 0.823, Pearson correlation, p<0.001; Figure 2).

## Discussion

Noroviruses are estimated to cause 23 million cases of gastroenteritis in the United States each year and to be the most common cause of foodborne gastroenteritis (*13*). Relatively few data describe the quantity and duration of fecal norovirus shedding as determined by modern assays. In a human experimental Norwalk virus infection model, we found that Norwalk virus could be detected in fecal samples for a median of 4 weeks and for up to 8 weeks after virus inoculation and that peak virus titers were most commonly found in fecal samples collected after resolution of symptoms. The peak virus titers (median 95 × 10^9^ copies/g feces) were higher than would be expected from electron microscopic studies (*5*,*14*). These observations help explain the epidemiologic observations of norovirus outbreaks linked to food handlers who had recovered from symptomatic infection (*15*) and in persons who had no gastroenteritis (*16*).

Only a few studies have used quantitative RT-PCRs to examine fecal viral load, and these studies have been primarily of GII norovirus strains. Chan et al. (*17*) described patients who shed >10^11^ norovirus copies/g feces, whereas the peak fecal virus titer observed by Ozawa et al. (*18*) in symptomatic and asymptomatic food handlers was ≈10-fold lower. Each of these studies was of persons with naturally acquired norovirus infection. However, the median peak viral load observed in our study (10^11^) was much higher than the 10^7^–10^8^ median viral loads reported in the prior studies (*17*,*18*). Lee et al. (*19*) noted higher viral loads in patients who had more prolonged symptoms (>4 days) associated with infection caused by GII.4 norovirus. Amar et al. (*20*) also reported viral loads to be higher in persons who had symptomatic gastroenteritis than in those who had been asymptomatic for at least 3 weeks. Our findings suggest that clinical gastroenteritis was associated with higher peak virus shedding and higher total virus shedding during the first 2 weeks after inoculation. Although we did not see an association of peak virus titer with symptom duration, the median duration of symptoms averaged only ≈1 day in our study. Potential reasons for the different results observed in other studies include the manner in which samples were collected (single samples vs. serial collection), the real-time assays used (generic assays designed to be broadly reactive vs. assay designed specifically for Norwalk virus detection), virulence of the infecting strains, differences in the populations studied (e.g., age, immune status), and the small number of infected persons who did not have clinical gastroenteritis in our study.

The development of more sensitive methods to detect noroviruses has been associated with a corresponding increase in the duration of recognized virus shedding (*1*,*8*). For example, Rockx et al. (*21*) found norovirus in fecal samples for >3 weeks in ≈25% of infected persons, and Murata et al. (*22*) found norovirus in fecal samples for up to 6 weeks in infected infants. In contrast, at least half of the participants in our study still had Norwalk virus in fecal samples after 4 weeks and 2 had virus still present at 8 weeks; we cannot exclude the possibility that these 2 persons shed for a longer period. Determination of whether the virus is still infectious must await the development of more sensitive and reproducible methods for norovirus cultivation than are currently available (*23*). 
